# Hairy Cell Leukemia Patients Have a Normal Life Expectancy—A 35-Year Single-Center Experience and Comparison with the General Population

**DOI:** 10.3390/cancers14051242

**Published:** 2022-02-28

**Authors:** Jan-Paul Bohn, Sabrina Neururer, Markus Pirklbauer, Andreas Pircher, Dominik Wolf

**Affiliations:** 1 Department of Internal Medicine V, Hematology and Oncology, Medical University of Innsbruck, A-6020 Innsbruck, Austria; andreas.pircher@i-med.ac.at (A.P.); dominik.wolf@i-med.ac.at (D.W.); 2 Department of Medical Statistics, Informatics and Health Economics, Medical University of Innsbruck, A-6020 Innsbruck, Austria; sabrina.neururer@i-med.ac.at; 3 Department of Internal Medicine IV, Nephrology and Hypertension, Medical University of Innsbruck, A-6020 Innsbruck, Austria; markus.pirklbauer@i-med.ac.at

**Keywords:** cladribine, purine analogues, interferon-alpha, long-term, overall survival

## Abstract

**Simple Summary:**

Classic hairy cell leukemia (HCL) is a rare, chronic B-cell malignancy with long-term remissions after standard treatment with purine analogues introduced in the 1990s. Although nearly all patiently ultimately relapse after up to 20 years from therapy requiring retreatment, overall survival prognosis remains insufficiently defined as life expectancyhas rarely been corrected for that of the general population. The aim of this single-center retrospective analysis was to report the long-term clinical outcomes of 83 consecutive HCL patients and compare overall survival with a matched cohort from the general population. We show that HCL patients may look forward to a normal lifespan when treated with purine analogues irrespectively of their pretreatment history, age at diagnosis, or whether they were treated until or after the year 2000.

**Abstract:**

Classic hairy cell leukemia (HCL) is an uncommon hematologic malignancy characterized by an excellent prognosis since purine analogues (PA), such as cladribine (2-CdA), have been introduced in the 1990s. However, most data on long-term outcomes is gathered from patients treated with PA first-line or include limited information on previous treatment outcomes, i.e., Interferon-α (IFN-α). Survival curves from previous series did not reach a plateau, indicating that nearly all patients ultimately relapse. Yet, overall survival (OS) data were rarely corrected for life expectancy of the general population. We here report 83 consecutive HCL patients treated between 1983 and 2017 at the University Center in Innsbruck, Austria. Median follow-up was 170 months (1–498). IFN-α, the first-line treatment of choice before 1990, was administered to 24 patients, achieving an overall response rate (ORR) of 86% and an unconfirmed complete remission (CRu) in 23%. All these patients relapsed after a median progression-free survival (PFS) of 30 months (3–80), but either remained drug-sensitive upon re-exposure to IFN-α or were successfully salvaged with PA. All 42 patients exposed to first-line 2-CdA responded (ORR of 100%). Sixteen patients received two to four successive courses of PA with a continuous decrease in the response quality (CRu rate 85.7% 1st-line vs. 41.5% 3rd-line treatment). Median PFS was not reached in both treatment-naïve patients and those retreated at first relapse. Although pretreatment with IFN-α was associated with a shortened median PFS of 81 months (43–118) after PA therapy, this tendency of inferior PFS did not result in inferior OS. OS of all 83 patients was excellent and equivalent to that of age-, sex-, and diagnostic period-matched controls from the Tyrolean general population (standardized mortality ratio 0.8), regardless of their age at diagnosis or whether they were diagnosed until or after the year 2000. These results confirm that HCL patients may look forward to a normal lifespan when treated with PA irrespective of their pretreatment history.

## 1. Introduction

Classic hairy cell leukemia (HCL) is a rare, indolent B-cell lymphoproliferative disorder characterized by progressive infiltration of the bone marrow (BM) and spleen, resulting in pancytopenia and infectious complications [1]. When the disease was first described in 1958 by Borouncle et al. [2], HCL patients were confronted with a dismal survival prognosis of less than five years. Only the introduction of purine analogues (PA), such as cladribine (2-CdA), in the 1990s enabled induction of long-lasting remissions, thereby altering the natural course of the disease [3,4,5]. However, most data on long-term outcome a gathered from patients treated with PA first-line or include limited information on previous treatment outcomes, i.e., with Interferon-α (IFN-α) [3,4]. Larger series often describe multi-center patient cohorts receiving heterogeneous supportive therapies, i.e., growth factor and infection prophylaxis [6]. Most importantly, in most retrospective analyses, survival curves did not reach a plateau, indicating that nearly all patients ultimately relapse. However, overall survival (OS) rates were rarely corrected for life expectancy in the general population [7].

We here present a 35-year retrospective, single-center experience from 1983–2017 reflecting clinical outcomes of 83 consecutive Tyrolean HCL patients after first- and subsequent lines of therapy and correlating survival with age-, sex-, and race-matched controls from the Tyrolean general population.

## 2. Methods

Between January 1983 and December 2017, consecutive HCL patients treated at a tertiary care cancer center in Innsbruck, Austria, were identified from the clinical database of Innsbruck University Hospital, and their outcomes were evaluated retrospectively. The study was approved by our institutional review board and by our ethics committee and was conducted in accordance with the Declaration of Helsinki in 1964 and its amendments. A total of 91 patients were given the diagnosis of HCL based on WHO criteria by morphological and flow cytometry analysis (FACS) of peripheral blood (PB) and BM.

Treatment was initiated only in patients with absolute neutrophil counts < 1.0 × 10^9^/L, hemoglobin concentration < 10 g/dL, platelet counts < 100 × 10^9^/L, and/or symptomatic splenomegaly. The choice of therapy was based on era-specific guidelines, ranging from splenectomy before introduction of IFN-α in 1984, which has been widely replaced by PA, such as 2-CdA, in the early 1990s. 2-CdA has been administered either intravenously or subcutaneously (0.14 mg/kg) for 5–7 consecutive days. 2-CdA combined with 4–6 weekly cycles of the anti-CD20 antibody rituximab (375 mg/m^2^ intravenously) was considered in patients with inferior PFS (<3 years) after PA-monotherapy. Novel targeted agents, such as the BRAF-inhibitor vemurafenib, the Bruton’s kinase inhibitor ibrutinib, as well as the novel anti-CD20 antibody Obinutuzumab, have been considered as salvage treatment in heavily pretreated patients as soon as they became available in clinical practice (starting with vemurafenib in 2012). Overall, eight HCL patients who have been treated with these novel drugs outside clinical trials at our center have been described in various case reports [8,9,10] and were excluded from this analysis to avoid recurrent reporting of recently published data. Relapsed/refractory disease to PA +/− CD20-antibody treatment and/or infectious complications due to pancytopenia guided treatment decisions with novel agents in these patients. All these eight HCL patients were known to be alive at the end of the observation period of this analysis. 

Safety and tolerability were reported by recording evidence, severity, and type of adverse events (AE) according to the National Cancer Institute Common Terminology Criteria for AEs v4.0.

During follow-up, patients were usually monitored for disease progression via physical examination and peripheral blood (PB) counts at 3, 6, 12, and 24 months after treatment initiation. Subsequently, PB counts and abdominal sonography were performed annually. Bone marrow evaluation after HCL diagnosis was only confined to cases with unclear cytopenia.

Responses were determined using standard response criteria. Unconfirmed complete remission (CRu) was defined as normalization of peripheral blood counts and resolution of organomegaly without BM biopsy performed to confirm response. Relapse after CRu was determined as reappearance of hairy cells in PB or BM, cytopenia, and/or splenomegaly on physical examination. Partial remission (PR) describes a > 50% improvement of cytopenias and organomegaly. Relapse after PR was defined as greater than 50% increase in residual disease. Patients not meeting any of these criteria were classified as non-responders (NR).

Categorial variables were summarized as frequencies and percentages and compared using Fisher’s exact test or chi-square test depending on their distribution. Presence or absence of normal distribution was assessed using the Kolmogorov–Smirnov test. Continuous variables were summarized as median values and range and compared using Mann–Whitney U test. Progression-free-survival (PFS) was defined for patients achieving a CRu or PR and was measured from the date of treatment initiation until first relapse or death from any cause. Observations of PFS were censored at date of last contact for patients with no report of relapse who were last known to be alive. Overall survival (OS) described the time from the date of diagnosis to the event of death. Patients last known to be alive were censored. PFS and OS estimates were determined by Kaplan–Meier analysis. OS and PFS were compared using the log-rank test. All these analyses were done using IBM SPSS Statistics 24 (New York, NY, USA) and GraphPad Prism 8 (San Diego, CA, USA).

Relative survival was defined as OS within the patient cohort divided by the expected OS from the general population. We matched our patients to individuals from a Tyrolean standard population with respect to age, sex, and diagnosis period. Ederer II method was used to calculate the expected OS. Relative survival was measured from the diagnosis date until death or end of follow-up. Survival was calculated for two calendar periods (≤2000, >2000) to better detect possible discrepancies in relative survival for HCL patients treated first-line with IFN-alpha or first-line with 2-CdA. We chose ≤2000 and >2000 as cut-off, as 2-CdA became generally available for Tyrolean HCL patients as soon as in the later 1990s (officially licensed on 10 October 1996). We calculated relative survival for two age categories (<60 years, ≥60 years) to better reflect possible discrepancies in treatment outcomes between younger and older/elderly HCL patients. The age threshold of 60 years was chosen in the absence of a clear definition of “older/elderly” HCL patients, and only few patients were over 70 years of age at diagnosis in our cohort. Statistical analyses were performed using Stata 16 (StataCorp. 2019. Stata Statistical Software: Release 16. College Station, StataCorp LLC, College Station, TX, USA).

## 3. Results

Our cohort includes 83 Tyrolean HCL patients with a median age at diagnosis of 54 years (range: 25–84 years) and a female-to-male ratio of 1:5 (Table 1). Eight patients were under 39 years, 65 patients between 40 and 69 years, and 10 patients were >70 years of age, respectively. Clinically, 66% of patients had splenomegaly, while 94% of the patients had at least cytopenia of one hematopoietic lineage in the peripheral blood counts (Table 1), with thrombocytopenia (84%) being most frequently detected. Anemia was seen in 72% of cases and neutropenia in 76% of cases.

**Figure 1:**
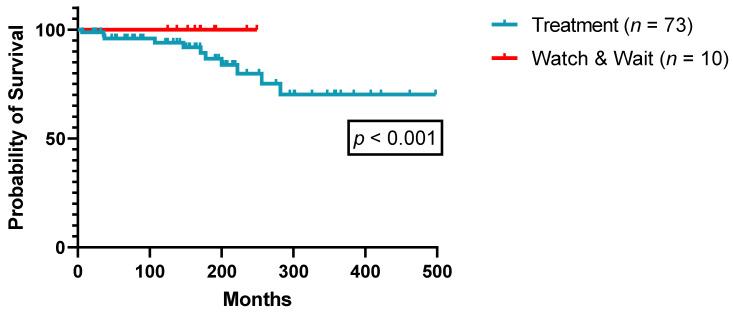
Overall survival according to treatment necessity (*n* = 83).

Eventually, 73/83 patients (88%) met the criteria for specific HCL treatment initiation, i.e., of PA and IFN-α, in the observation period, with IFN-α being the agent of choice before 1990 and PA thereafter (Table 2). A total of 10/83 patients (12%) did not meet the criteria for treatment initiation as outlined in the methods section and were carefully monitored for disease progression (“Watch & Wait”). All these 10 patients were diagnosed after the year 2000 and known to be still alive at the end of the observation period. Figure 1 illustrates OS comparison between treated patients and those not meeting the criteria for treatment initiation during the observation period.

### 3.1. Treatment with Interferon-α

IFN-α was administered front-line to 24 patients, with a median treatment duration of 13 months (range: 1–36 months) and an ORR of 87.5% (CRu 20.8%, PR 66.7%, Table 3). IFN-α was given at varying dosages (4.5–9 × 10^6^ U/week) adjusted according to toxicity and disease control and was prematurely discontinued due to side effects (fatigue) in two patients. All 24 IFN-α-exposed patients relapsed after a median PFS of 30 months (range: 3–80 months). IFN-α rechallenge was performed in 37.5% of cases, and 15 patients diagnosed before 1992 received even multiple courses of IFN-α, e.g., in one patient, up to seven cycles, inducing long-term disease control for 25 years. Another previously splenectomized patient prone to frequent infectious complications resulting from profound neutropenia was repeatedly bridged with IFN-α to PA treatment, allowing adequate disease control for over 30 years [11]. Thirteen of all twenty-four relapsed patients were switched from IFN-α to second-line treatment with PA.

### 3.2. Treatment with Purine Analogues +/− Rituximab

**Figure 2:**
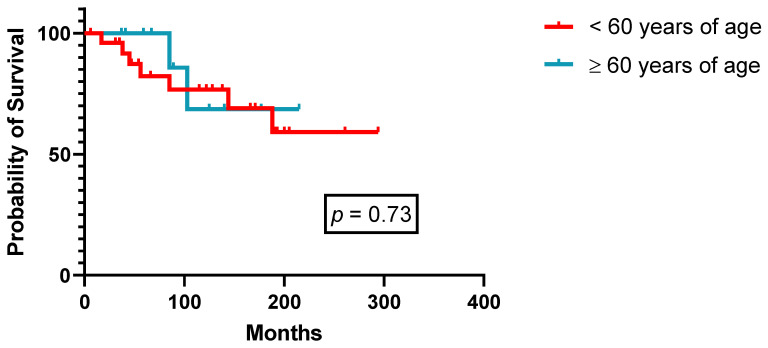
Progression-free survival after first-line 2-CdA according to age at diagnosis (*n* = 42).

**Figure 3:**
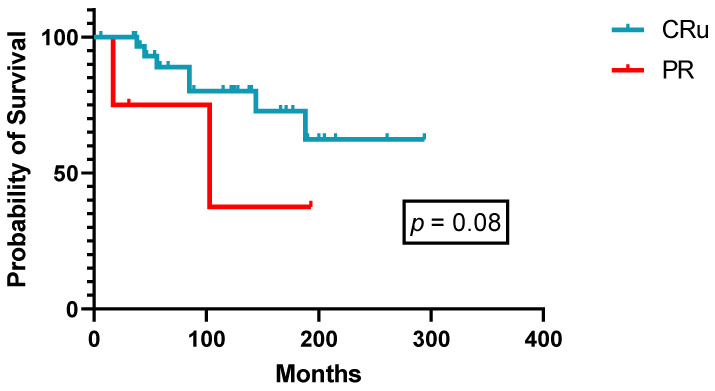
Progression-free survival after first-line 2-CdA according to response (*n* = 42).

Forty-two patients received 2-CdA as first-line therapy (Table 2) with an ORR of 100% (CRu 85.7%, PR 14.3%, Table 3), of which 90% of courses were administered after 1994. With a median follow-up of 152 months (range: 1–347), median PFS was not reached in patients both under 60 years of age at diagnosis (*n* = 27) or above (*n* = 15, Figure 2). Although not reaching statistical significance, there is a tendency for superior PFS in patients achieving a CRu (*p* = 0.08, Figure 3). All nine relapsing patients received another course of 2-CdA, and second-line median PFS was not reached as well. Over the years, 16 patients received two to four successive courses of PA, mostly 2-CdA, with a steady decrease in terms of quality of responses (CR rate 85.7% 1st-line versus 41.5% 3rd-line treatment, Table 3). Three patients were given 2-CdA in combination with rituximab (375 mg/m^2^ intravenously, weekly × 4–6), as second-, third-, and fifth-line treatment, respectively, due to inferior PFS (<3 years) after 2-CdA monotherapy (Table 2). Two patients achieved a CRu and one patient a PR ongoing for 8, 3, and 7 years, respectively. Two of them experienced a cytokine release syndrome grade 2 during the first rituximab infusion.

Overall, 66 HCL patients (79.5%) were treated with PA during the observation period. 

Febrile neutropenia was the most commonly reported severe adverse event, which was reported in 14 patients (33.3%) treated with PA front-line, in 7 patients (32%) at first relapse, and 4 patients (30.4%) treated third-line with PA. Febrile neutropenia did not correlate with treatment outcome. A total of 38 (90.5%) treatment-naïve and 8 (88.9%) retreated patients received infection prophylaxis with valaciclovir and co-trimoxazole. G-CSF prophylaxis was given to 49 patients (74.2%) and did not seem to impact the occurrence of febrile neutropenia. Median time to recovery of neutrophils was not significantly different in patients with (36 months, range: 15–89 months) or without G-CSF prophylaxis (33 months, range: 11–76 months, *p* > 0.05) or whether PA was given as front-line (32 months, range: 21–71 months) or second-line (25.5 months, range: 21–79 months, *p* > 0.05) treatment. Secondary malignancies occurred in 11 patients (16.7%) treated with PA, of whom one patient died of acute myeloid leukemia in 2012 after having received three cycles of 2-CdA in 1993, 1999, and 2006, respectively. Other malignancies were: three adenocarcinomas of the prostate, two rectal adenocarcinomas, one diffuse large B-cell non-Hodgkin lymphoma, one malignant melanoma, one pancreatic adenocarcinoma, one urothelial adenocarcinoma, and one gastric adenocarcinoma.

### 3.3. Comparison of Purine Analogues and Interferon-α

**Figure 4:**
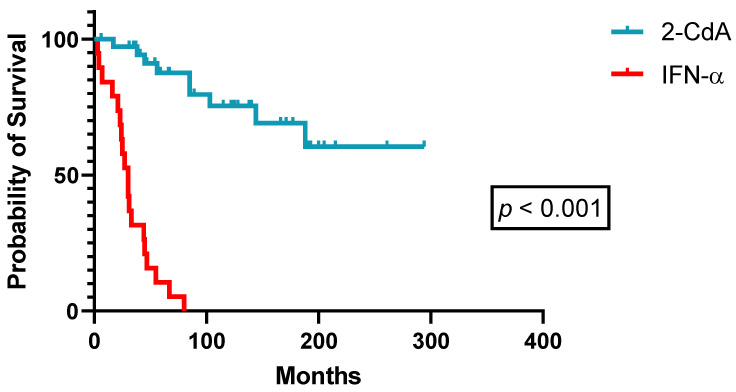
Progression-free survival after first-line therapy (*n* = 66).

**Figure 5:**
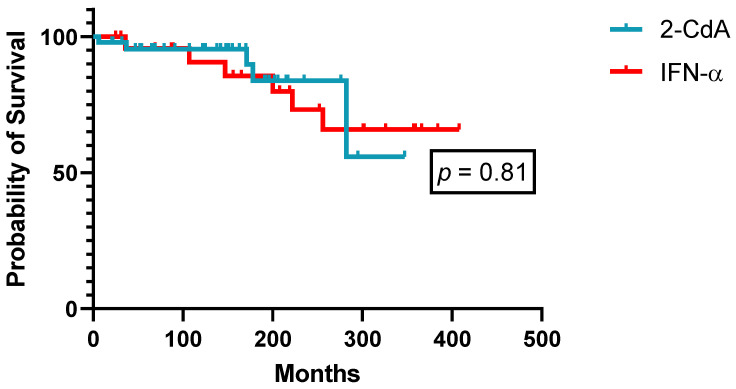
Overall survival according to first-line therapy (*n* = 66).

A detailed overview of demographics and patient characteristics treated front-line with either IFN-α or PA is given in Table 4. Besides a significantly longer follow-up for patients treated with IFN-α front-line (treatment of choice before 1990), patients’ baseline demographics did not reveal significant discrepancies between both treatment groups. In treatment-naïve HCL patients, 2-CdA was superior to IFN-α in terms of quality (85.7% versus 20.8% CRu, *p* < 0.01, Table 3) and duration of responses (median PFS: not reached versus 30 months, *p* < 0.001, Figure 4). Twenty-four patients initially treated with splenectomy or IFN-α eventually received next-line treatment with PA. Efficacy was similar when PA was used as second-line treatment in these patients (ORR 100%, CR 81.8%, PR 18.2%), but median PFS stayed confined to 81 months (range: 43–118 months). This tendency towards a shorter PFS with second-line PA in pretreated patients with splenectomy and/or IFN-α did not translate into inferior OS, as successive PA retreatment in accordance with universally accepted guidelines achieved sufficing long-term disease control. As such, OS curves of patients treated front-line with either IFN-α or 2-CdA did not separate (Figure 5).

### 3.4. Comparison with the Austrian General Population

**Figure 6:**
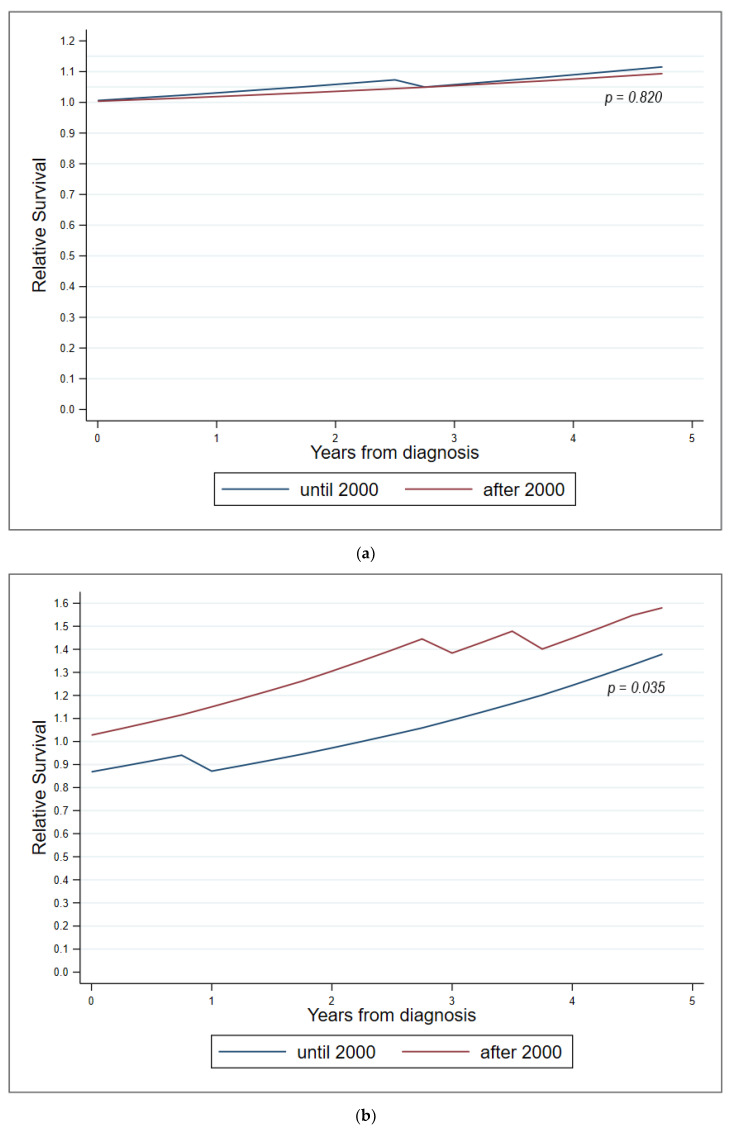
Survival comparison with a matched cohort from the Tyrolean general population. (**a**) Patients < 60 years; (**b**) patients ≥ 60 years.

OS prognosis after diagnosis of our 83 HCL patients was equivalent to that of an age-, sex-, and diagnostic period-matched cohort from the Tyrolean general population. The overall standardized mortality ratio (SMR, observed-to-expected ratio) was 0.8 (95% confidence interval, CI 0.6–1.0). This positive trend was consistent for both female (SMR = 0.8, 95% CI 0.6–1.0) and male patients (SMR = 0.9, 05% CI 0.5–1.6) and persisted for patients both under and above the age of 60 years at diagnosis (Figure 6). Relative survival of HCL patients under 60 years of age compared to a matched cohort from the Tyrolean general population was similar for those diagnosed until and after the year 2000 (*p* = 0.82). In contrast, relative survival of HCL patients above 60 years of age at diagnosis was superior when the patients were diagnosed after the year 2000 compared to those diagnosed before (*p* = 0.035). The trend for relative survival advantage increased with time after diagnosis for all of the described subgroups (Figure 6).

## 4. Discussion

Reviewing 35 years of clinical experience with HCL patients, this is one of the largest single-center analysis reflecting on both therapeutic eras in terms of IFN-α and PA usage before and after the 1990s, respectively. Almost one-third of patients in our series were treated first-line with IFN-α at varying dosages adjusted according to toxicities and disease control. We report a high ORR (87.5%) and relatively low CRu rate (20.8%) similar to efficacy data documented in a recent analysis [12]. Without additional myelotoxicity, no infection prophylaxis was given, and no infectious complications were reported. All of these patients eventually experienced relapse but remained drug-sensitive when re-challenged, allowing long-term disease control. All relapsing patients treated with PA (mainly 2-CdA) achieved a CRu but showed limited median PFS when compared to patients pre-treated first-line with PA. This tendency of shorter PFS when pretreated with IFN-α may likely be a result of low patient numbers (*n* = 13) and did not result in inferior OS, as PA retreatment in accordance with universally accepted guidelines achieved sufficing long-term disease control. Herein, our long-term analysis illustrates that IFN-α still remains an effective and safe therapeutic alternative when PA treatment is considered not to be the optimal therapy, i.e., in patients with a tendency for infectious complications, and may not entail inferior clinical outcomes, particularly as all patients can be “salvaged” by subsequent PA treatment in case of disease relapse.

In more recent years, 2-CdA evolved as treatment standard [13]. Thus, one-half of this patient cohort were treated front-line with 2-CdA either as two-hour infusion for 5 days or as subcutaneous application for 5 days. Overall, 100% ORR, >86% CRu rates, and an unreached median PFS after a median follow-up of nearly 13 years underscore its role as treatment of choice for HCL in the front-line and relapsed setting and reflect recent efficacy data [14,15]. Febrile neutropenia is the most frequent complication of PA therapy [16]. However, in our patients G-CSF prophylaxis and infection prophylaxis did not impact either the incidence of febrile neutropenia or the median time to recovery of neutropenia. These results seem concordant with a recent retrospective study investigating the impact of prophylactic versus “on demand” filgastrim in 159 2-CdA-treated HCL patients. The authors demonstrated similar rates of infections, need for hospitalization, and nadir duration in both cohorts, suggesting insufficient benefit for prophylactic G-CSF administration [17]. Accordingly, both approaches seem justifiable and are chosen at the physicians discretion.

Sixteen of our patients received up to four courses of 2-CdA with similar clinical outcomes, albeit with a tendency of decreased response quality and duration, nevertheless providing long-term disease control. Equally important to note, however, is the substantial proportion of patients not requiring any further retreatment after front-line 2-CdA. Twenty patients remained free of treatment for more than ten years, nine patients for more than fifteen, and three patients for more than twenty years, respectively. Herein, our data are in line with previous analyses suggesting that 2-CdA may indeed “cure” the disease in individual HCL patients in terms of achieving an excellent long-term leukemia clearance [18], although it remains elusive how to identify this subpopulation at diagnosis and/or treatment initiation. Sigal and colleagues [19] analyzed 19 HCL patients with confirmed ongoing CR after a median of 16 years from a single course of 2-CdA and found minimal residual disease (MRD) in 37% (7/19) of cases as assessed by BM immunochemistry; 16% of patients even had morphologic evidence of HCL. On the other hand, Lopez and co-workers [20] reported longer median treatment-free survival in 42 MRD-free HCL patients (not reached) compared to 40 MRD-positive HCL patients (97 months, range: 38–156 months; *p* = 0.059). With these conflicting results with regard to its clinical significance and consequence in mind, detection of MRD as identified by immunochemistry or immunophenotyping analysis of BM has not found entrance into clinical practice yet [21]. Of interest, however, 8-color flow cytometry monitoring of HCL cells in PB may allow identification of patients at high risk for clinical relapse. In a French study of 34 HCL patients in CR after treatment with 2-CdA, MRD detection (>10^−4^ HCL cells) resulted in a significantly higher risk of clinical relapse as compared to the MRD-negative individuals (1/9 patients with undetectable MRD versus 5/6 patients with detectable MRD; *p*= 0.003) [22]. As recently demonstrated in a comparative prospective phase 2 study, 2-CdA with concurrent rituximab may enhance both the rate of undetectable MRD as well as durability of MRD negative remissions [23]. Lacking significant PFS benefits with short follow-up and potentially associated with more profound myelotoxicity, however, combination treatment has not entered commonly accepted treatment recommendations in the front-line setting [24]. Given the clinically indolent nature of the disease, long-term follow up and OS corrected for the life expectancy in the general population may indeed serve as more reliable variable to reflect treatment outcomes.

In this respect, a large, population-based analysis in the Netherlands recently reported that nowadays HCL patients < 70 years of age treated with PA monotherapy may experience a normal life expectancy, raising the question of whether treatment intensification may generally be advisable and beneficial [25]. In the here-presented Tyrolean cohort of 83 HCL patients, we found a nearly equivalent OS prognosis compared to age-, sex-, and period-matched controls of the Tyrolean general population. Remarkably, the positive trend in OS prognosis was seen in HCL patients both under and over 60 years of age. Whereas HCL patients under 60 years of age were to expect a similar relative OS compared to the matched cohort from the Tyrolean general population throughout the entire observation period, HCL patients above 60 years of age showed a significant improvement in relative OS when diagnosed after the year 2000 (*p* = 0.035). These survival benefits pronounced in the elderly may partially be a result of selection bias, as HCL patients more frequently encounter the national health systems for disease monitoring than individuals from the general population do, who only rely on preventive health check-ups recommended for healthy Tyroleans. Thus, emerging age-related comorbidities may have been detected and treated earlier than in the matched general population. 

In summary, our results suggest that HCL patients may look forward to a normal lifespan when treated with PA irrespective of their pretreatment history, age at diagnosis, or whether they were treated until or after the year 2000. Limitations of the present study are the retrospective, single-center design as well as the rather small sample size. The single-center approach, however, also enabled life-long monitoring of a homogeneously treated patient population in terms of drug dosing (i.e., IFN-α), infection prophylaxis, and G-CSF support.

## 5. Conclusions

With these excellent long-term outcomes in mind, accumulating toxicity rather than inadequate disease control appears to become the limiting factor in improving HCL patients’ clinical outcome. Herein, several early clinical trials investigating chemotherapy-free approaches with BRAF- and MEK-inhibitors with or without the addition of an anti-CD20-antibody report encouraging clinical efficacy and good tolerability [26,27,28,29]. Ultimately, these novel combinations with targeted drugs may increasingly supersede the current and long-standing top position of PA.

## Figures and Tables

**Table 1:** Patient characteristics (*n* = 83).

Median Age at Diagnosis, Years (Range)	54 (25–84)
Females (%)	14 (17)
Follow-up (months), median (range)	170 (1–498)
Splenomegaly, *n* (%)	55 (66)
Longitudinal diameter in ultrasound, cm (range)	16 (14–30)
Neutrophil count (×10^9^/L), median (range)	0.8 (0.1–1.4) (*n* = 42)
Hemoglobin (g/L), median (range)	119.5 (54–170)
Platelet count (×10^9^/L), median (range)	79 (19–438)
Any cytopenia, *n* (%)	78 (94%)
BRAFV600 mutation verified	21 (25%, 100% after 2012)

cm, centimeter.

**Table 2:** Details of Treatment Lines.

Lines (1–5)	1st Line	2nd Line	3rd Line	4th Line	5th Line	Patients (*n*)
1	2-CdA					42
	IFN-α					24
	Splenectomy					7
2	2-CdA	2-CdA				8
	2-CdA	2-CdA + R				1
	IFN-α	2-CdA				11
	IFN-α	IFN-α				9
	IFN-α	Pentostatin				2
	Splenectomy	IFN-α				6
	Splenectomy	Chlorambucil				1
3	2-CdA	2-CdA	2-CdA + R			1
	IFN-α	2-CdA	2-CdA			5
	IFN-α	IFN-α	IFN-α			4
	IFN-α	IFN-α	2-CdA			3
	Splenectomy	IFN-α	Pentostatin			3
	Splenectomy	IFN-α	IFN-α			2
	Splenectomy	IFN-α	2-CdA			1
	Splenectomy	Chlorambucil	IFN-α			1
4	IFN-α	IFN-α	IFN-α	2-CdA		2
	IFN-α	IFN-α	IFN-α	IFN-α		1
	IFN-α	IFN-α	2-CdA	2-CdA		1
	IFN-α	2-CdA	2-CdA	2-CdA		1
	Splenectomy	IFN-α	IFN-α	Pentostatin		1
	Splenectomy	IFN-α	Pentostatin	Pentostatin		1
	Splenectomy	IFN-α	Pentostatin	IFN-α		1
	Splenectomy	Chlorambucil	IFN-α	Pentostatin		1
5	IFN-α	IFN-α	IFN-α	IFN-α	IFN-α	1
	IFN-α	IFN-α	2-CdA	2-CdA	2-CdA	1
	IFN-α	2-CdA	2-CdA	IFN-α	2-CdA	1
	IFN-α	2-CdA	2-CdA	IFN-α	2-CdA + R	1
	Splenectomy	IFN-α	Pentostatin	IFN-α	2-CdA	1

*n*, number; 2-CdA, cladribine; IFN-α, interferon-α, R, rituximab.

**Table 3:** Response after first and subsequent lines of treatment with IFN-α or purine analogues.

Response	IFN-α			2-CdA/Pentostatin		
	1st Line (*n* = 24)	2nd Line (*n* = 15)	3rd Line (*n* = 7)	1st Line (*n* = 42)	2nd Line (*n* = 20)	3rd Line (*n* = 12)
ORR (%)	87.5	80.0	57.2	100	100	91.5
CRu (%)	20.8	13.3	14.3	85.7	90	41.5
PR (%)	66.7	66.7	42.9	14.3	10	50
NR (%)	12.5	-	-	-	-	8.3

IFN-α, interferon-α; 2-CdA, cladribine; Cru, undetermined complete remission; ORR, overall response rate; PR, partial remission; NR, no response.

**Table 4:** Comparison of patient characteristics according to first-line treatment with 2-CdA or IFN-α.

Patient Characteristics	2-CdA (*n* = 42)	IFN-α (*n* = 24)	*p*-Value
Median age at diagnosis, years (range)	53.5 (30–84)	53.5 (25–74)	0.61
Females (%)	9 (21)	1 (4)	0.08
Follow-up (months), median (range)	152 (1–347)	220 (25–408)	0.01
Splenomegaly, *n* (%)	31 (75)	24 (56)	0.44
Longitudinal spleen diameter in ultrasound, cm (range)	16 (12–30)	17 (16–29)	0.36
Neutrophil count (×10^9^/L), median (range)	0.8 (0.1–1.4)	not evaluable	not applicable
Hemoglobin (g/L), median (range)	119 (59–170)	96.5 (54–128)	0.08
Platelet count (×10^9^/L), median (range)	75 (19–328)	73 (58–303)	0.83
Any cytopenia, *n* (%)	35 (83)	19 (79)	0.30

2-CdA, cladribine; IFN-α, Interferon-α; cm, centimeter.

## Data Availability

The data presented in this study are available on request from the corresponding author. The data are not publicly available due to data protection.
